# Endocannabinoids and Precision Medicine for Mood Disorders and Suicide

**DOI:** 10.3389/fpsyt.2021.658433

**Published:** 2021-05-20

**Authors:** Graziano Pinna

**Affiliations:** The Psychiatric Institute, Department of Psychiatry, University of Illinois at Chicago, Chicago, IL, United States

**Keywords:** endocannabinoid system, suicide, biomarkers, depression, PTSD, animal model

## Introduction

Diagnostic precision, prediction and prevention of psychiatric disorders, including major unipolar depression, post-traumatic stress disorder (PTSD) and suicide ideation and attempts, remain underdeveloped areas in psychiatry given a general lack of biomarker assessment in the field. This makes the unmet goal of developing a precision medicine for these debilitating conditions an urgent necessity of neuropsychopharmacology research. Precision medicine, defined as “an emerging approach for treatment and prevention that takes each person's variability in genes, environment, and lifestyle” into account (1), will permit choosing the right treatment for the right person at the right time based on a unique individual neurobiologic biosignature. Indeed, it is anticipated that biomarker discovery will tremendously enhance refinement of individualized medicine that currently rely on subjective symptom assessment based on the Diagnostic and Statistical Manual of Mental Disorders, 5th Edition (DSM-V). Both depression and PTSD are highly prevalent conditions affecting 3.4–12% of the general population and one of the main causes of disability. In the United States, pre-Covid suicide rates have increased by 25–30%, (from 10.5 to 13 per 100,000). These disorders share a number of symptoms and are highly comorbid. MDD is characterized by sadness, anhedonia, disturbed concentration, while PTSD symptoms include avoidance of traumatic memories, hyperarousal, hyperreactivity, flashbacks and nightmares. Antidepressant treatment with SSRIs (the gold standard for PTSD and depression) improves symptoms to about half of patients (2). Developing reliable biomarkers entails the promise of predicting the best treatments for subjects that are more likely to respond to an individually-designed rather than to a “one-fit-all” treatment. Biomarker discovery will also enhance diagnostic evaluation of patients who suffer from psychiatric disorders that share a large symptom overlap and prevalent disorder comorbidity. In recent years, several novel biomarker candidates for mood disorders have been suggested [reviewed in (3)]. The endocannabinoid system has received much interest owing its role in several physiological and pathophysiological functions, including regulation of emotional behavior, cognitive processes, inflammation, chronic pain, epilepsy, and in general, its role underlying neuropsychiatric disorders (4, 5).

This opinion article will focus on the intriguing role of the endocannabinoid system in the regulation of affective disorders, specifically on major depressive disorders, PTSD and suicide behaviors. Furthermore, it will analyze whether data gathered in this exciting area of psychiatric research entails new leads in establishing novel biomarkers for these debilitating and prevalent psychiatric conditions that affect millions worldwide.

## The Endocannabinoid and Endocannabinoid-Like Systems and Stress Response

**Figure 1:**
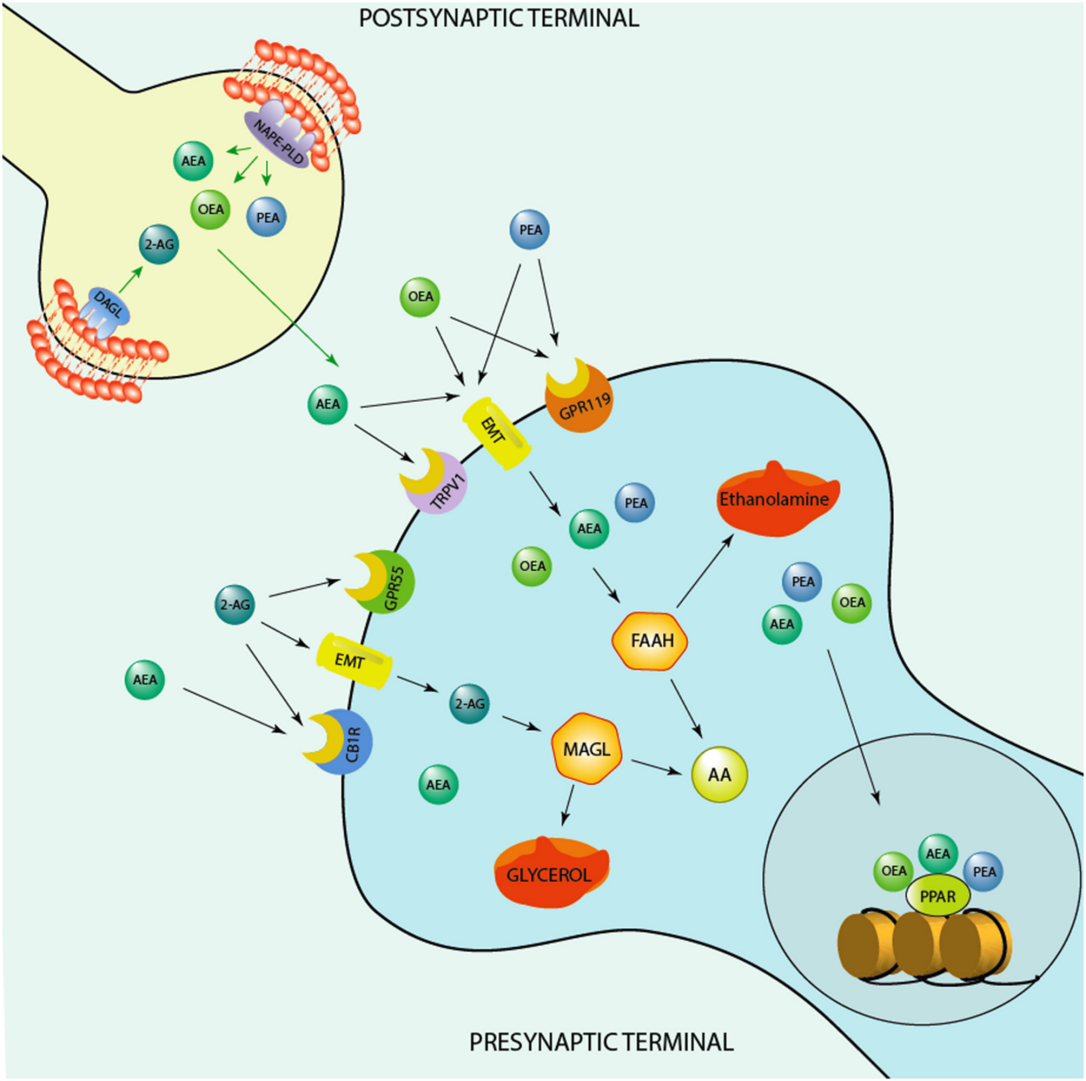
Schematic representation of the endocannabinoid system. Depicted are several biosynthetic and degradation pathways as well as endocannabinoid receptors that are involved in the action of the endocannabinoids, anandamide (AEA), 2-Arachidonoylglycerol (2-AG), and of the endocannabinoid-like ethanolamines, oleoylethanolamide (OEA) and N-palmitoylethanolamine (PEA). AEA, PEA, and OEA share the similar biosynthetic pathway after originating from membrane's phospholipids are synthesized post-synaptically by the action of the enzyme, N-acylphosphatidylethanolamine-specific phospholipase D (NAPE-PLD). 2-AG is instead produced by the action of the enzyme, diacylglycerol lipase (DAGL), prior to be secreted by post-synaptic terminals and act at pre-synaptic cannabinoid receptor type 1 (CB1) and G protein-coupled receptor 55 (GPR55). AEA, PEA, and OEA can act at membrane receptors or be taken up pre-synaptically through endocannabinoid membrane transporters (EMT). They can be degraded by the action of the enzyme, fatty acid amide hydrolase (FAAH) into ethanolamine and arachidonic acid (AA) pre-synaptically. These endocannabinoids influence each concentration by competing for the catalytic action of FAAH. For instance, increased levels of AEA can compete for the catalytic action of FAAH and thereby result in an increase of PEA and OEA levels or vice versa, PEA and, mostly, OEA by competing for FAAH catalytic action may increase AEA levels. PEA may also decrease FAAH expression and thereby elevate its own and the levels of OEA and AEA. 2-AG is instead degraded by monoacylglycerol lipase (MAGL) to glycerol and AA. While OEA and PEA fail to bind to the classic CB1 and CB2, they can influence the action of AEA at transient receptor potential channels of vanilloid type-1 (TRPV1). PEA may activate peroxisome proliferator-activated receptor-alpha (PPAR-α) as well as TPRV1. What makes the endocannabinoid system attractive for developing novel biomarkers concerns the fact that it is constituted by several components, including synthesizing and degrading enzymes to receptors and endogenous modulators and it is widely distributed in the brain. These neuromodulators are implicated in several mechanisms that regulate neuronal functions, including cognition and emotional behavioral regulation. Likewise, synthetic agents that stimulate endocannabinoid receptors or act on the degrading/biosynthetic enzyme constitute a valid pharmacological approach for treatment of several neuropsychiatric disorders. For instance, the action of AEA binding at CB1 and of PEA at PPAR-α has been associated with a fast improvement of emotional behavioral deficits, including aggressive behavior and impulsivity (11, 12), which are behavioral endophenotypes of human behavioral-traits of suicide risk. In humans, studies show higher CB1 and CB1-mediated G-protein activation in the dorsolateral prefrontal cortex (DLPFC) of suicide victims (13). Studies conducted in alcoholic suicide victims have evidenced enhanced CB1 activation and increased AEA and 2-AG concentrations in the DLPFC (14). Furthermore, CB1 expression was increased in the ventral striatum of suicide individuals who struggled with alcoholism (15). Intriguingly, both FAAH expression and activity was found upregulated in post-mortem brain of suicide subjects (15). Together, these findings underlie profound deficits within the endocannabinoid system. More studies are warranted to understand the precise role of endocannabinoid levels, their biosynthetic enzymes as well as their receptors (CB1 and PPAR-α) in suicide victims.

The endogenous cannabinoid system includes the widely investigated anandamide (AEA), that acts as a partial agonist for the cannabinoid receptor type 1 (CB1) and type 2 (CB2) (6), and 2-arachidonoyl-glycerol (2-AG), which acts as a full agonist for both these receptors (7). Both endocannabinoids are synthesized and released from post-synaptic terminals and traffic retrogradely to act at presynaptic CB1/CB2 receptors (8). The biosynthetic enzymes involved in their production and metabolism are the fatty acid amide hydrolase (FAAH) for AEA (9) and monoacylglycerol lipase (MAGL) for 2-AG (10) (Figure 1). CB1 is heavily expressed in brain areas devoted in the regulation of stress responses and emotions, which include the prefrontal cortex, ventral hippocampal regions and the basolateral amygdala (16). Mechanistically, CB1 and CB2 receptors inhibit the presynaptic release of neurotransmitters, including GABA and glutamate (17, 18). This action has notoriously been associated with the regulation of anxiety exerted by endogenous and synthetic cannabinoids. In preclinical studies, several CB1 agonists show anxiolytic effects (19), however, this anxiety-like pharmacological effect show a bimodal action, becoming anxiogenic at higher doses (20). Intriguingly, increasing the levels of AEA by genetic deletion of FAAH or using pharmacological FAAH inhibitors (URB597) ameliorates anxiety-like behavior (21). This finding is supported by data showing treatment with rimonabant (SR141716), a selective CB1 inhibitor, increases anxiety and depression (22).

In addition to AEA and 2-AG, endocannabinoid-like modulators include the ethanolamine-derivative N-palmitoylethanolamine (PEA) and its congener, oleoylethanolamide (OEA) (23). PEA is produced by the biosynthetic action of the enzyme N-acyl phosphatidylethanolamine phospholipase D (NAPE-PLD), and, like AEA, is metabolized by FAAH and, more specifically, by N-acylethanolamine acid amide hydrolase (NAAH) (23). PEA is the endogenous modulator of the transcription factor/nuclear receptor, peroxisome proliferator-activated receptor (PPAR)-α, that after heterodimerizing with retinoid X receptor-α, modulates the expression of target genes (24). Like CB1, PPAR-α is expressed throughout the brain, including hippocampus, amygdala and prefrontal cortex (25), and implicated in a host of physiological and pathological processes, including, neuronal differentiation, inflammation, mitochondrial and proteasomal dysfunction, oxidative stress, and neurodegeneration (26).

Stress affects the endocannabinoid system and metabolite levels in opposite directions. While acute stress increases 2-AG, it reduces AEA by enhancing FAAH activity (27–29). Accordingly, preclinical studies show that chronic stress reduces the concentrations of AEA in the amygdala-hippocampal-cortico-striatal circuit (30). These findings support the notion that these endocannabinoids are implicated in distinct neurobiological processes. The role of PEA and PPAR-α on stress response is less studied and understood. However, evidence shows that stress induces a fast FAAH activation resulting in AEA and PEA level reductions (27, 29). PEA levels also decrease when rodents are exposed to predator stress –a model of PTSD (31), and increase after short-term stress in humans (32). Similarly to fluoxetine, administration with PEA induces antidepressant pharmacological effects (11, 33) and pharmacological inhibition of PEA degradation or its biosynthesis upregulation also yields improvement of depressive-like behavior (34–36).

## Role of Endocannabinoids in Mood Disorders and Suicide

The endocannabinoid system has been implicated in the neuropathophysiology of stress-related neuropsychiatric disorders (37), however, the role of endocannabinoids in mood disorders is sparse and limited. Among individuals with PTSD, evidence shows a dysregulation in the endocannabinoid signaling. For example, reduced levels of AEA are linked with depression and PTSD (20, 38) and a down-regulation of peripheral AEA levels is associated with an up-regulation of CB1 in brain (39). A genetic polymorphism in the human gene encoding FAAH is implicated in the dysregulation of FAAH-mediated AEA hydrolysis. This drives to a peculiar endophenotype that is associated with reduced index of trait anxiety and enhanced cortico-amygdala connectivity (40, 41). Clinical studies also show the involvement of an abnormal function of the endocannabinoid system in suicide subjects. For instance, evidence shows higher CB1 and CB1-mediated G-protein activation in depressed suicide dorsolateral prefrontal cortex (DLPFC) (13). These findings were also mirrored by studies of alcoholic suicide victims that have evidenced elevated CB1 activation and increased AEA and 2-AG levels in the DLPFC (14). Hence, these similarities between depressed suicide and alcoholic suicide victims point to a role for the endocannabinoid system in suicide in alcoholism and depression. Other studies have showed that CB1 expression is elevated in the ventral striatum of alcohol-dependent suicide subjects (15). Both FAAH expression and activity increased in suicide post-mortem brain (15), which underlay profound abnormalities of the endocannabinoid system. The observation that elevated CB1–mediated signaling in DLPFC of depressed subjects who died by suicide together with the elevated levels of endocannabinoids and CB_1_ receptor function strongly supports a hyperactive endocannabinoid system. Whether these are adaptation mechanism remains to be further clarified. However, in some post-mortem studies that include comorbidity with suicide, it is challenging to prove a given neurobiological parameter is linked to the pathophysiology of suicide alone.

In a cross-sectional study comparing morning serum concentrations of AEA, 2-AG but also that of the endocannabinoid-like congeners, PEA and OEA in 30 suicide attempters and 12 psychiatric controls found that, in the morning, AEA and PEA serum levels were increased in suicide attempters compared to controls, unrelated of cannabis use. When cannabis use was controlled in the urine and accounted in the analyses, AEA and PEA serum concentrations still remained elevated. This study supports a role for AEA and PEA in the pathophysiology of suicidal behavior. However, this limited study should be expanded and replicated in larger cohorts (42).

In preclinical studies, deletion of the gene encoding CB1 induces aggressive behavior in male mice following the exposure of a same-sex conspecific “intruder” in their home cage –a behavioral trait of suicide-like behavior (43). Interestingly, a later study conducted by the same group shows the relevance of CB2 receptors in the development of the suicidal-like phenotype in mice. CB2-KO mice present higher levels of aggressive behavior both in the social interaction and the resident intruder paradigms compared to wild-type mice (43).

The content of PEA was found altered in several diseases and disorders, which include multiple sclerosis, traumatic brain injury, chronic pain, neuroinflammation, and various neurodegenerative diseases (23). Notwithstanding its role and that of its congeners remains largely underinvestigated in psychiatric disorders, recent studies observed that PEA, OEA, and stearoylethanolamide (SEA) levels are significantly reduced in male and female patients in a manner that correlated with severity of PTSD symptoms (44). This finding is in line with preclinical studies that have showed that PEA concentrations were elevated following antidepressant treatment in corticolimbic areas of rodents (45) and that administration of PEA improves fear extinction and anxiety-like behaviors, a pharmacological action that is abolished in PPAR-α-KO mice or after administration with PPAR-α antagonists (11). In depressed patients, PEA increases the pharmacological efficacy of the antidepressant citalopram in improving depressive symptoms (46). These observations are further supported by studies showing that physical exercise exert a strong antidepressant effect and this action correlated with enhancement of AEA, PEA, and OEA levels in PTSD and MDD subjects (47).

## The Potential Role of the Endocannabinoid System as a Biomarker of Mood Disorders and Suicide

The endocannabinoid system is a neuromodulatory system among the most expressed in human and rodent brain and implicates the action of several other neurotransmitter systems, including the GABAergic, glutamatergic, and serotonergic, for the most part. Its role in the regulation of emotions has significantly advanced our understanding of the pathophysiological mechanisms leading to mood disorders. Developing reliable biomarkers for mood disorders remains one urgent goal in molecular psychiatry so that patients at risk can be timely protected by highly debilitating conditions, such as major unipolar depression and PTSD that are highly comorbid with suicide. This relies on establishing animal models that closely mirror these prevalent stress-induced pathological conditions and establishing sophisticated technology to achieve this goal.

The summary above substantiates the concept that the endocannabinoid system is a novel and potential target underlying the neurobiology of mood disorders and suicide and may serve to exploit new treatments. Indeed, both preclinical and clinical studies show that the CB1 receptor and the endocannabinoids, AEA and 2-AG may play a role in suicide behaviors. Recent studies also suggest a role for the PPAR-α receptor and its endogenous modulators, PEA, OEA and SEA in PTSD and depression and in aggressive behavior and impulsivity in animal models of these mood disorders (12, 44). A better characterization of these systems would benefit the field of neuropsychopharmacology to better comprehend the endocannabinoid role in the mechanisms of mood disorders and suicide pathophysiology. This will also facilitate designing more efficacious preventive strategies to anticipate suicidal attempts.

Suicide is a rather complex psychiatric disorder that remains poorly understood and likely involving several neurotransmission systems, neuropeptides and neurohormones in addition to the role played by the endocannabinoid system. Evidence shows that PPAR-α engages the biosynthesis of the GABAergic neurosteroid, allopregnanolone to modulate emotional behavior, including fear responses and aggressive behavior (11, 12). Importantly, allopregnanolone is implicated in the pathophysiology of PTSD and depression and the US FDA has recently approved it as the first specific treatment for the treatment of post-partum depression (48). Hence, investigation on the neuronal circuitry and functional crosstalk between the endocannabinoid system and the neurosteroid biosynthesis may unveil more precise neurobiological targets underlying mood disorders and comorbid suicidal behaviors that may prove essential in developing novel therapeutic target for the treatment of these conditions.

New knowledge on the role of the endocannabinoid system in human pathophysiology has been allowed by quantifying serum/plasma endocannabinoids in patients with several neuropsychiatric conditions by gold standard technology. Some studies have explored endocannabinoids and related N-ethanolamines in saliva and studied how they change in relation to various pathophysiological conditions. For example, fasting plasma and salivary levels of endocannabinoids were quantified through liquid chromatography-mass spectrometry (LC-MS). While no studies have investigated the levels of the endocannabinoids, 2-AG and AEA, and their congeners OEA and PEA in blood vs. saliva in psychiatric disorders, these endocannabinoids were reliably quantifiable in saliva obtained by obese subjects. Their levels were significantly higher in obese than in normal subjects suggesting that salivary endocannabinoid levels might represent a useful biomarker in obesity (49). Novel investigations should address whether endocannabinoid levels assayed by state-of-the-art technology, including GC-MS or LC-MS, that provide unsurpassed structure selectivity and sensitivity, may correlate in blood and saliva and whether they also predict severity of psychiatric symptoms.

## Author Contributions

The author confirms being the sole contributor of this work and has approved it for publication.

## Conflict of Interest

The author declares that the research was conducted in the absence of any commercial or financial relationships that could be construed as a potential conflict of interest.
